# Characteristics and Clinical Significance of Intestinal Microbiota in Patients with Chronic Hepatitis B Cirrhosis and Type 2 Diabetes Mellitus

**DOI:** 10.1155/2022/1826181

**Published:** 2022-05-13

**Authors:** Xiu Sun, Xin Chi, Yingying Zhao, Shunai Liu, Huichun Xing

**Affiliations:** ^1^Center of Liver Diseases Division 3, Beijing Ditan Hospital, Capital Medical University, Beijing, China; ^2^National Center for Infectious Diseases, Beijing, China; ^3^Beijing Key Laboratory of Emerging Infectious Diseases, Institute of Infectious Disease, Beijing Ditan Hospital, Capital Medical University, Beijing, China; ^4^Peking University Ditan Teaching Hospital, Beijing, China

## Abstract

**Background:**

Chronic hepatitis B cirrhosis is often accompanied by glucose metabolism disorder, and intestinal microbiota was closely related to both cirrhosis and diabetes. There are few studies on the role of intestinal microbiota in hepatitis B liver cirrhosis and diabetes mellitus (LCDM). The purpose of this study was to investigate the characteristics of intestinal microbiota in patients with LCDM and to evaluate the relationship between the severity of intestinal microbiota imbalance and clinical significance.

**Methods:**

A case-controlled study was conducted. People who met the inclusion and exclusion criteria of chronic HBV-related liver cirrhosis (LC), LCDM, and healthy controls (HC) were enrolled in, and their fecal and blood samples were collected. The V3-V4 region of 16s rDNA gene of fecal microbiota was sequenced; the bioinformatics analysis including *α*-diversity, *β*-diversity, and linear discriminant analysis (LDA) effect size (LEfSe) was performed; and the correlation between bacteria and clinical indexes was analyzed.

**Results:**

A total of 70 participants completed fecal and blood tests, including 20 HC, 20 LCDM, and 30 LC. The *α* diversity of intestinal microbiota in the LCDM decreased than that in the HC. The abundance of *Proteobacteria*, *Streptococcus*, *Escherichia-Shigella*, and *Lactobacillus* increased, while the abundance of *Bacteroidota*, *Bacteroides*, *Prevotella*, *Faecalibacterium*, and *Lachnospira* decreased in the LCDM compared with the HC. The abundance of *Lactobacillus*, *Roseburia*, and *Veillonella* and the degree of hepatitis B cirrhosis dysbiosis indicator (HBCDI) increased in the LCDM than in the LC. The abundance of *Escherichia-Shigella*, *Veillonella*, and *Lactobacillus* positively correlated with liver injury and fasting blood glucose (FBG) level. The abundance of *Escherichia-Shigella*, *Veillonella*, *Streptococcus*, and *Lactobacillus* increased more significantly when FBG and glycosylated hemoglobin level increased.

**Conclusion:**

Intestinal microbiota of patients with LCDM was significantly disordered, and the degree was more serious than that cirrhosis patients without diabetes.

## 1. Introduction

Hepatitis B virus (HBV) infection is a global health problem, and HBV-associated cirrhosis is one of the major causes of death worldwide [1]. The liver plays a key role in glucose homeostasis. Patients with chronic liver diseases are often accompanied by glucose metabolism disorders; up to 30% of patients with cirrhosis suffer from diabetes mellitus (DM) [2, 3]. HBV-related liver disease and DM interact with each other [4]. On the one hand, liver diseases related to HBV infection can insult blood glucose metabolism and induce insulin resistance through various mechanisms [4, 5]. On the other hand, the complication of DM may increase the risk of decompensation of liver disease with chronic hepatitis B [6]. DM is an independent factor for the deterioration of cirrhosis and is associated with the occurrence of major complications, including ascites, renal insufficiency, hepatic encephalopathy, and bacterial infection [7]. It also increases the risk of hepatocellular carcinoma (HCC) in patients with chronic HBV infection [8].

Recent studies found that the intestinal microbiota was closely related to chronic hepatitis B cirrhosis and DM [9–11]. In hepatitis B cirrhosis, the diversity of intestinal microbiota decreased, and the increase in the abundance of *Escherichia coli* and *Veillonella* positively correlated with the Child–Turcotte–Pugh (CTP) score, while the decrease in the abundance of *Bacteroides* negatively correlated with the CTP score [12]. The change in intestinal microbiota affected the occurrence and development of liver disease [11, 12]. In DM, the diversity of intestinal microbiota decreased, and butyric acid–producing bacteria were significantly related to insulin resistance or type 2 diabetes [13]. Therefore, this study investigated whether characteristic changes in intestinal microbiota occurred in LCDM and how these changes were related to the disease progression, thus laying a foundation for exploring new strategies for the diagnosis and treatment of liver diseases from the perspective of intestinal microecology.

## 2. Materials and Methods

### 2.1. Study Population

This was a case-controlled study approved by the Medical Ethics Committee of Beijing Ditan Hospital, Capital Medical University (DT-IRB-2018-04001). All participants were required to sign the informed consent to participate in the study. The study was executed following the standards recommended by the Good Clinical Practice (GCP) guidelines.

Patients with a diagnosis of hepatitis B cirrhosis were eligible for the study if they met the following criteria:

- They were over 18 years old
- Their HBsAg positive and/or HBV-DNA positive were more than 6 months
- Their imaging examination suggested cirrhosis, portal hypertension, esophageal varices, splenomegaly, with or without ascites, without infection, hepatic encephalopathy, and gastrointestinal bleeding

Patients with a diagnosis of LCDM were eligible for the study if they met the following criteria:

- They met the aforementioned diagnostic criteria for hepatitis B cirrhosis
- They were previously diagnosed with type 2 diabetes or had more than two random blood glucose ≥ 11.1 mmol/L or fasting blood glucose (FBG) ≥ 7.0 mmol/L at different times

The exclusion criteria were as follows: patients complicated by liver diseases such as alcoholic liver disease, autoimmune liver disease, fatty liver disease, and other viral liver diseases; patients who had systematically used antibiotics, probiotics, and proton pump inhibitors within 1 month before enrollment; and pregnant and lactating women.

In addition, patients whose results of physical examination, blood routine, urine routine, liver function, kidney function, serological markers of HBV, and abdominal ultrasound were within the normal range, and those without heart, brain, kidney, and lung diseases, were selected as healthy controls (HC).

### 2.2. Data Collection and Specimen Collection

All participants eligible for inclusion were required to provide demographic information and previous health status and record the eating habits for 1 month earlier. The fasting peripheral venous blood was collected on the day of enrollment. Also, fresh fecal samples were collected in a sterile stool retention box and transferred to a –80°C refrigerator for storage within half an hour.

### 2.3. Physiological Index Detection

All blood samples were tested in the laboratory of Beijing Ditan Hospital, Capital Medical University, on the day of collection. The liver functions, such as alanine aminotransferase (ALT), aspartate aminotransferase (AST), albumin (ALB), total bilirubin (TBil), renal function (creatinine), and FBG, were examined using an automatic biochemical analyzer (Hitachi, 7600-020). The blood routine, such as white blood cells (WBC), red blood cells (RBC), blood platelets (PLT), and hemoglobin (HGB), was examined using an automatic blood cell analyzer (Sysmex, XN-10). The prothrombin time was examined using an automatic blood coagulation analyzer (Werfen, ACLTOP750CTS). The serum markers of HBV (hepatitis B surface antigen, hepatitis B surface antibody, hepatitis B e antigen, and so on) were analyzed using an automatic chemiluminescence analyzer (Beckman Coulter, ACCESS2). The HBV DNA load was examined using a gene amplification instrument and its supporting reagent (Roche, Cobas).

### 2.4. Gene Extraction and Sequencing of Fecal Samples

Total genomic DNA was extracted from the fecal samples by the cetyltrimethylammonium bromide method. According to the selection of the sequencing region (the 16S V3–V4 region), the barcode-specific primers (the primer was 341F-806R: 341F-CCTAYGGGRBGCASCAG, 806R-GGACTACNNGGGTATCTAAT) were used for polymerase chain reaction amplification. The PCR products were purified by agarose gel electrophoresis with a concentration of 2%, and the sequences with the main strip of 400–450 bp were chosen for further experiments. The sequencing libraries were generated using an Illumina TruSeq DNA PCR-Free Library Preparation Kit (Illumina, USA). The library quality was assessed with a Qubit@ 2.0 Fluorometer (Thermo Scientific) and an Agilent Bioanalyzer 2100 system. At last, the library was sequenced on an Illumina NovaSeq platform.

### 2.5. Bioinformatics and Statistical Analysis

The *α* diversity index (Chao1 index and ACE index) was calculated using the QIIME software (version 1.9.1). The *β* diversity was used binary Jaccard distance principal coordinate analysis to evaluate the microbial structure and distribution. Venn diagram was drawn using the R software (version 2.15.3). The linear discriminant analysis (LDA) effect size (LEfSe) was used to screen different species between different groups. A log LDA score > 2 was the threshold of differential taxa.

The chi-square test, nonparametric test, and Student *t*-test were used to compare the categorical variables and continuous variables between the groups. A *P* value < 0.05 indicated a statistically significant difference.

## 3. Results

### 3.1. Clinical Characteristics of Enrolled Participants

**Figure 1:**
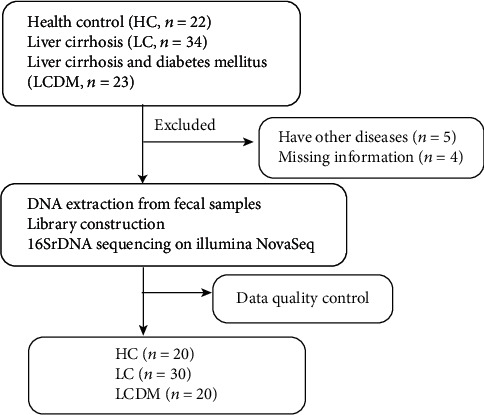
Research design and flow chart. A total of 79 stool samples were collected from Beijing Ditan Hospital, Capital Medical University. After strict inclusion and exclusion process, 70 samples were finally enrolled in, including 20 cases of healthy controls (HC), 30 cases of HBV-related liver cirrhosis (LC), and 20 cases of hepatitis B liver cirrhosis and diabetes mellitus (LCDM). The characteristics of intestinal microbiota were analyzed of the included participants.

A total of 79 samples were screened out, of which 5 complicated with other diseases and 4 with missing information were excluded. Finally, 70 enrolled participants completed the detection of 16SrDNA in blood and feces, including 20 cases of HC, 30 cases of LC, and 20 cases of LCDM (Figure 1). No infection, hepatic encephalopathy, and gastrointestinal bleeding were found in patients with LC and LCDM. The Child-Pugh scores of A, B, and C were 8, 19, and 3 cases (26.7%, 63.3%, and 10%) in LC and 6, 12, and 2 cases (30%, 60%, and 10%) in LCDM, respectively. No difference in severity of liver diseases between LC and LCDM (*P* = 0.966). In LC and LCDM, 22 patients (73.3%) and 14 patients (70%) received antiviral treatment for more than one year, and 8 patients (26.7%) and 6 patients (30%) received antiviral treatment for less than one year, respectively. There is no significant difference in antiviral treatment of LC and LCDM (*P* = 0.797). In LCDM, five patients (25%) were treated with insulin, four (20%) were treated with acarbose, one (5%) was treated with insulin combined with acarbose, one (5%) was treated with metformin, and nine (45%) controlled blood glucose through diet and exercise. The blood glucose levels at the time of enrollment are shown in Table 1. The demographic characteristics and clinical data of the participants are shown in Table 1. No significant differences were found in age, sex, and body mass index (BMI) among the three groups (*P* > 0.05). The FBG level in the LCDM group was significantly higher than that in the LC and HC groups (*P* < 0.05). Also, no significant differences were observed in the levels of AST and ALB, WBC counts, RBC counts, HGB level, PLT counts, and HBeAg between the LCDM and LC groups (*P* > 0.05). Although not all patients had HBV DNA load lower than the lowest detection limit, there was no significant difference in viral load between LC and LCDM group. Therefore, it indicated that the intestinal microbiota difference between the two groups was mainly due to the presence or absence of diabetes.

### 3.2. Intestinal Microbiota Characteristics in Patients with LCDM

**Figure 2:**
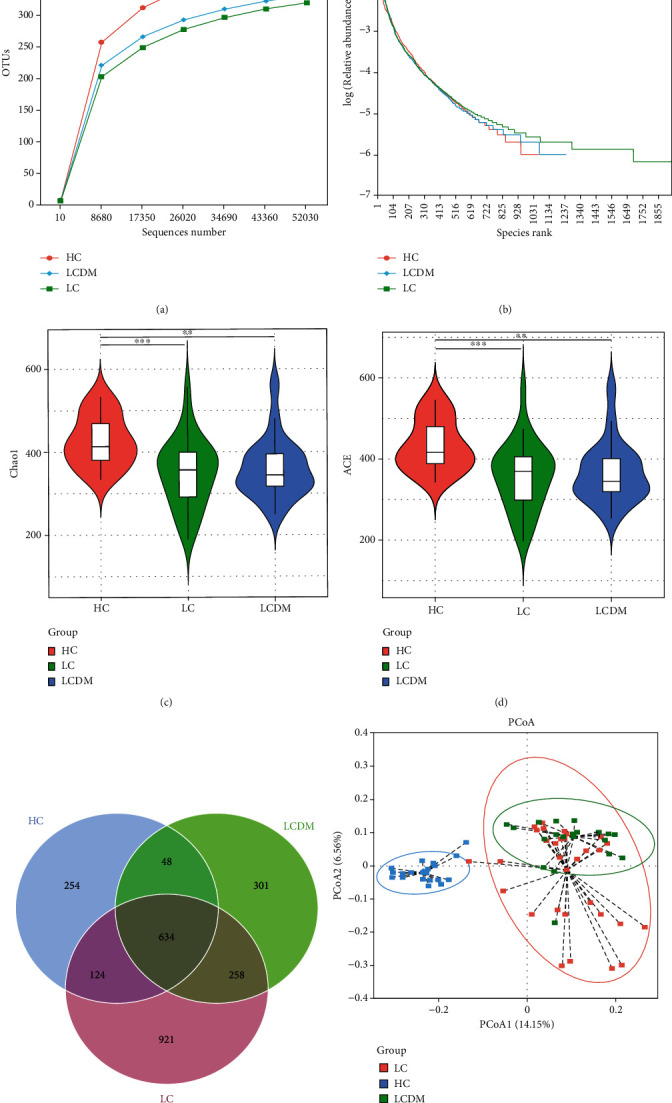
Comparison of intestinal microbiota diversity among HC, LC, and LCDM. (a) The rarefaction curve tends to be flat, indicating that the sequencing depth was sufficient. (b) The rank abundance curve tends to be flat, indicating even species distribution. (c) Chao1 index and (d) ACE index significantly decreased in LCDM. (e) The Venn diagram showed that 892 OTUs were shared between LC and LCDM, and 301 OTUs were unique to LCDM. (f) The *β* diversity of PCoA analysis based on binary Jaccard distance showed that the samples of intestinal microbiota in the HC group were clustered and were farther away from those in the LC and LCDM groups, while the spatial distance between samples in the LC and LCDM groups was less. HC: healthy controls; LC: HBV-related liver cirrhosis; LCDM: hepatitis B liver cirrhosis and diabetes mellitus.

All the samples were clustered into 2675 OTUs, including 33 phyla, 59 classes, 153 orders, 260 families, and 563 genera. The rarefaction curve and the rank abundance curve in each group tended to be flat, indicating that the sequencing depth was sufficient and the sample species distribution was uniform (Figures 2(a) and 2(b)). The *α* diversity of intestinal microbiota (Chao1 index and ACE index) decreased in the LC and LCDM groups compared with the HC group, but no significant differences were found between the LC and LCDM groups (Figures 2(c) and 2(d)). The Venn diagram showed that 634 OTUs were shared between the 3 groups, 892 OTUs were shared between the LC and LCDM groups, and 301 OTUs were unique to the LCDM group (Figure 2(e)). The *β*-diversity of intestinal microbiota was calculated based on binary Jaccard distance principal coordinate analysis. It showed that the samples of intestinal microbiota in the HC group were clustered and were farther away from those in the LC and LCDM groups, while the spatial distance between samples in the LC and LCDM groups was less, but still different (Figure 2(f)).

**Figure 3:**
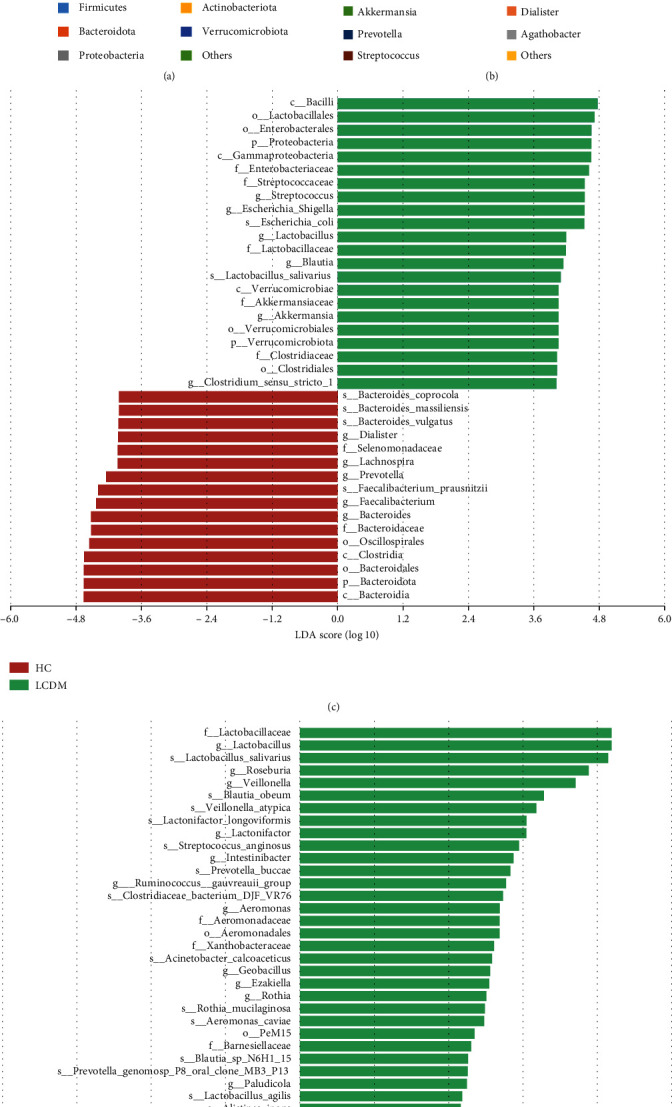
Characteristics of intestinal microbiota of HC, LC, and LCDM. The relative abundance of intestinal microbiota in HC, LC, and LCDM at the phylum (a) and genus level (b). (c) Differential taxa identified by LEfSe analysis in HC and LCDM (LDA > 4.0). (d) Differential taxa identified by LEfSe analysis in LC and LCDM (LDA > 2.0). HC: healthy controls; LC: HBV-related liver cirrhosis; LCDM: hepatitis B liver cirrhosis and diabetes mellitus.

At the phylum level, the relative abundance of *Proteobacteria* in the HC, LC, and LCDM groups was 2.97%, 13.75%, and 11.97%; the relative abundance of *Bacteroidota* was 38.29%, 22.75%, and 29.47%; and the relative abundance of *Actinobacteriota* was 5.82%, 9.30%, and 3.86%, respectively (Figure 3(a)). At the genus level, the abundance of *Streptococcus* and *Lactobacillus* increased gradually, and the abundance of *Faecalibacterium* and *Agathobacter* decreased gradually. The relative abundance of *Escherichia-Shigella* among the three groups was 0.77%, 7.78%, and 7.5%, respectively (Figure 3(b)). The characteristics of intestinal microbiota in different groups were analyzed by the LDA effect size (LEfSe) method. Compared with the HC group, *Proteobacteria* was the dominant phylum in the LCDM group, and the relative abundance of *Streptococcus* (LDA = 4.53, *P* = 3.67*e* − 07), *Escherichia-Shigella* (LDA = 4.53, *P* = 4.84*e* − 06), *Lactobacillus* (LDA = 4.19, *P* = 7.0*e* − 06), *Blautia* (LDA = 4.14, *P* = 6.24*e* − 05), and *Akkermansia* (LDA = 4.05, *P* = 0.009) increased, while the relative abundance of *Bacteroidota*, *Bacteroides*, *Faecalibacterium*, *Prevotella*, and *Lachnospira* decreased in the LCDM group (Figure 3(c)). Despite no significant difference in diversity, the bacterial composition was different between the LC and LCDM groups. The relative abundance of *Lactobacillus* (LDA = 4.19, *P* = 0.0075), *Roseburia* (LDA = 3.88, *P* = 0.002), *Veillonella* (LDA = 3.708, *P* = 0.0089), and *Lactonifactor* (LDA = 3.046, *P* = 0.0296) increased while the relative abundance of *Megasphaera* decreased in the LCDM group compared with the LC group (Figure 3(d)).

### 3.3. Relationship between Intestinal Microbiota and Clinical Indicators

**Figure 4:**
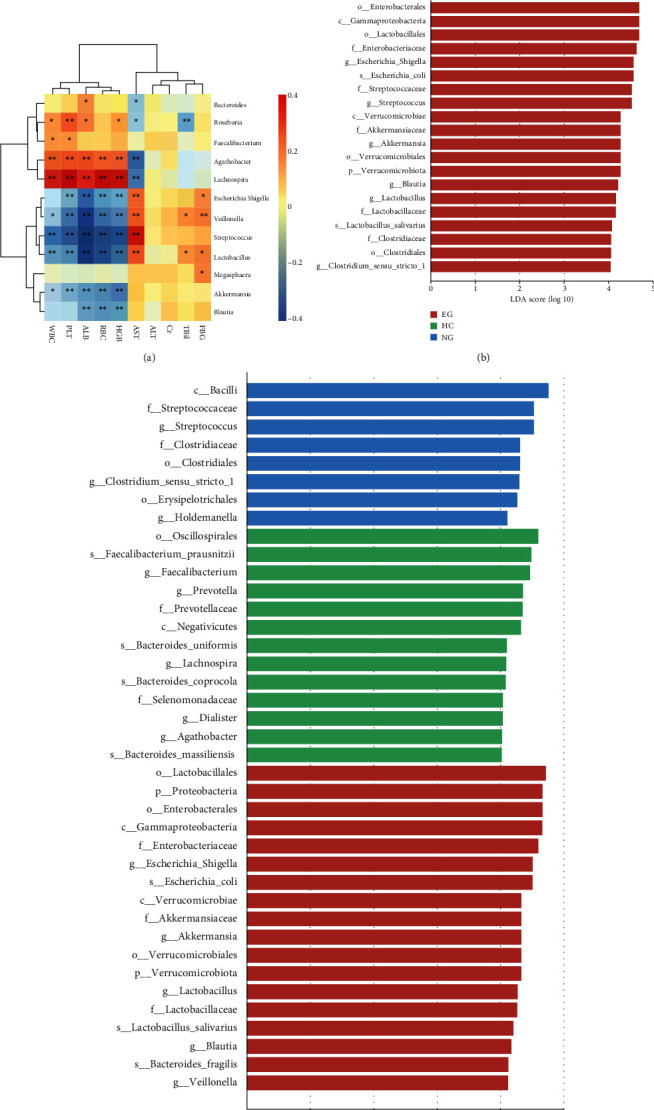
Correlation of intestinal microbiota with clinical indicators. (a) Intestinal microbiota were closely related to multiple clinical indicators. (b) Differential taxa identified by LEfSe analysis in HC, NG, and EG (LDA > 4.0). (c) Differential taxa identified by LEfSe analysis in HC, NH, and EH (LDA > 4.0). HC: healthy controls; NG: normal blood glucose; EG: elevated blood glucose; NH: normal glycosylated hemoglobin; EH: elevated glycosylated hemoglobin.

A correlation analysis was performed between different bacteria and clinical indicators to better understand the relationship between intestinal microbiota and clinical indicators. At the genus level, the abundance of *Escherichia-Shigella*, *Veillonella*, *Streptococcus*, and *Lactobacillus* positively correlated with the liver injury index AST (*r* = 0.337, *P* = 0.0044; *r* = 0.349, *P* = 0.003; *r* = 0.530, *P* = 2.38*e* − 06; *r* = 0.386, *P* = 0.001) and negatively correlated with PLT, ALB level, RBC count, and HGB (all *P* < 0.05). The abundance of *Escherichia-Shigella*, *Veillonella*, *Lactobacillus*, and *Megasphaera* also positively correlated with the FBG level (*r* = 0.266, *P* = 0.026; *r* = 0.306, *P* = 0.010; *r* = 0.298, *P* = 0.012; *r* = 0.295, *P* = 0.013). It indicated that the abundance of *Escherichia-Shigella*, *Veillonella*, and *Lactobacillus* correlated with not only liver injury indicators but also the FBG level (Figure 4(a)). In addition, the abundance of *Roseburia*, *Agathobacter*, and *Lachnospira* positively correlated with WBC count, PLT count, ALB level, and HGB level but negatively correlated with the AST level (all *P* < 0.05) (Figure 4(a)).

For patients with LCDM, the subgroup analysis was performed according to the patients' FBG level at the time of enrollment, including the normal blood glucose group (NG, *n* = 5) and the elevated blood glucose group (EG, *n* = 15). From the HC, the NG, to the EG group, the abundance of *Bacteroidota* tended to decrease, while the abundance of *Verrucomicrobiota* and *Proteobacteria* tended to increase (Supplementary Figure 1). The relative abundance of *Lactobacillus*, *Akkermansia*, *Escherichia-Shigella*, *Veillonella*, and *Streptococcus* showed an increasing trend, while the abundance of *Faecalibacterium*, *Prevotella*, *Bacteroides*, and *Lachnospira* showed a decreasing trend (Supplementary Figure 2). The LEfSe analysis (LDA > 4.0) also showed that *Proteobacteria*, *Escherichia-Shigella*, *Streptococcus*, *Akkermansia*, and *Lactobacillus* were significantly enriched in the EG group compared with the other groups (Figure 4(b)).

The subgroup analysis of patients with LCDM was performed according to the level of glycosylated hemoglobin at the time of enrollment; the patients were divided into a normal glycosylated hemoglobin group (NH, *n* = 7) and an elevated glycosylated hemoglobin group (EH, *n* = 13). From the HC, the NH, to the EH group, the abundance of *Verrucomicrobiota*, *Proteobacteria* (Supplementary Figure 3), *Akkermansia*, *Lactobacillus*, *Escherichia-Shigella*, and *Veillonella* showed an increasing trend, while the abundance of *Prevotella* showed a decreasing trend (Supplementary Figure 4). LEfSe analysis (LDA > 4.0) showed that *Streptococcus* was enriched in the NH group, while *Escherichia-Shigella*, *Akkermansia*, *Lactobacillus*, *Veillonella*, and *Blautia* were enriched in the EH group (Figure 4(c)). A close relationship was found between intestinal microbiota and the blood glucose level in patients with LCDM, and the imbalance of intestinal microbiota was obvious when the blood glucose level was poorly controlled.

The degree of intestinal microbiota imbalance in patients with LCDM was evaluated using the HBCDI. HBCDI = (*Escherichia* − *Shigella* + *Streptococcus* + *Lactobacillus*)/(*Ruminococcus* + *Prevotella* + *Bacteroides*) [14]. The median of HBCDI in HC, LC, and LCDM was 0.004, 0.176, and 0.403, respectively (*P* < 0.001), indicating that the imbalance of intestinal microbiota increased in patients with LCDM.

## 4. Discussion

A large number of bacteria are present in the human intestine; it is estimated that the gene of intestinal bacteria in the human body is 150 times that of the host gene [15]. Under normal circumstances, the intestinal bacteria participate in various physiological processes of the host, including intestinal mucosal barrier, energy, and immunity; they play an important role in the human body [16, 17]. The intestinal microbiota also plays an important role in regulating glucose metabolism [18]. In liver cirrhosis, intestinal biological disorders, intestinal barrier damage, and immune response changes occur. Intestinal bacterial products can translocate to liver through portal vein, where they are recognized by specific receptors, activate the immune system, lead to proinflammatory response, promote the occurrence and development of liver cirrhosis, and promote the disorder of glucose and lipid metabolism. On the other hand, portal hypertension in cirrhosis leads to intestinal edema, destruction of epithelial integrity, and more bacterial translocation [19]. LCDM has the both characteristics of liver cirrhosis and disorders of blood glucose metabolism, so we speculate that the intestinal microbiota may also play an important role in LCDM, which may be different from simple liver cirrhosis. Thus, we explored the intestinal microbiota characteristics of LCDM, thereby laying a foundation for understanding the pathological mechanism and potential treatment strategies of LCDM.

Compared with the HC group, the diversity and the relative abundance of *Faecalibacterium*, *Bacteroide*s, *Prevotella*, and *Lachnospira* decreased in the LCDM group, all of which were butyric acid–producing bacteria [20, 21], which was similar to that in patients with diabetes without cirrhosis [22, 23]. In addition, the abundance of *Escherichia coli*, *Streptococcus*, and *Lactobacillus* increased in prediabetes and diabetes mellitus [23–26]. In this study, the relative abundance of *Escherichia-Shigella*, *Streptococcus*, and *Lactobacillus* increased in the LCDM group; the abundance of these bacteria positively correlated with the liver injury index. The abundance of *Escherichia-Shigella* and *Lactobacillus* also positively correlated with the FBG level. A recent study has shown that *Escherichia coli*-derived LPS can promote blood glucose disorder and adipose inflammation, delay intestinal glucose absorption, and increase the secretion of insulin and glucagon-like peptide [27]. The probiotics in the intestinal microbiota decreased, and the abundance of potentially pathogenic bacteria increased in the LCDM group, which aggravated the imbalance of intestinal microbiota and was closely related to the blood glucose level. Therefore, the blood glucose level of patients with liver cirrhosis needs to be actively controlled. The abundance of *Roseburia* and *Akkermansia* reduced in diabetes. However, in our study, the abundance of these two bacteria increased in the LCDM group. Some studies suggested that metformin treatment of diabetes increased the relative abundance of *Akkermansia* [28]. We analyzed our participants; 55% of patients accepted hypoglycemic drugs such as acarbose, insulin, or metformin, which might contribute to the increase in the abundance of *Roseburia* and *Akkermansia*.

Another interesting finding of our study was that despite no difference in the diversity, still, some differences existed in the composition of intestinal microbiota between the LC and LCDM groups. The relative abundance of *Lactobacillus*, *Roseburia*, and *Veillonella* increased in the LCDM group compared with the LC group. This was consistent with the findings of Ke et al. [29]. In our previous study, the abundance of *Roseburia* and *Veillonella* significantly correlated with hepatitis B-related liver cancer. *Veillonella* could decompose lactic acid to produce a short-chain fatty acid propionic acid [30] and also produced lipopolysaccharide [31], thus triggering the LPS-TLR signaling pathway and promoting the progression of liver disease, which might be related to the virulence gene contained in this bacterium. *Roseburia* is a butyric acid–producing bacterium [32]. Usually, butyric acid is beneficial to the body, but it may also promote the occurrence of tumors in some circumstances [33]. Diabetes is an independent risk factor for HCC in patients with chronic HBV infection. The abundance of liver cancer-related bacteria in patients with LCDM increases, suggesting that the change of intestinal microbiota may be an important factor for the susceptibility of HCC in patients with LCDM.

In our previous study, we constructed HBCDI, an index reflecting the degree of intestinal microbiota imbalance in patients with hepatitis B cirrhosis, to assess the severity of the liver disease. In this study, the HBCDI was used to evaluate the degree of intestinal microbiota imbalance. The study found that the intestinal microbiota in patients with LCDM caused more serious injury than that in patients with LC, which indicated an increase in the total number of potential pathogens. Therefore, we should pay more attention to the microbiota of patients with LCDM. Probiotics may be beneficial to blood glucose homeostasis, reduce pathological bacterial translocation in liver cirrhosis [34], and improve immune response and liver function [35]. Therefore, this study shed light on the role of intestinal microbiota and the strategy of targeting intestinal microbiota treatments against LCDM.

## Figures and Tables

**Table 1:** Clinical characteristics of the enrolled participants.

Clinical indicators	HC (*n* = 20)	LC (*n* = 30)	LCDM (*n* = 20)	*P* value (HC vs. LCDM)	*P* value (DC vs. LCDM)
Gender (F/M)	6/14	5/25	4/16	0.465	1.0
Age	51.20 ± 8.43	54.53 ± 8.15	55.32 ± 6.47	0.104	0.929
BMI (***x̄*** ± *S*)	23.07 ± 3.20	24.10 ± 2.43	23.79 ± 4.12	0.583	0.614
ALT (U/L)	23.0 ± 10.36	24.49 ± 14.58	44.62 ± 95.04	0.758	0.566
AST (U/L)	19.79 ± 4.17	33.49 ± 11.64	55.28 ± 106.65	≤0.001	0.566
ALB (g/L)	44.90 ± 2.42	34.01 ± 5.61	31.79 ± 7.08	≤0.001	0.586
TBil (*μ*mol/L)	14.93 ± 3.91	27.69 ± 18.26	22.16 ± 17.28	0.183	0.169
WBC (10^9^/L)	5.03 ± 0.90	2.98 ± 1.65	3.37 ± 1.87	≤0.001	0.630
RBC (10^12^/L)	4.86 ± 0.34	3.71 ± 0.69	3.38 ± 0.76	≤0.001	0.078
HGB (g/L)	146.70 ± 12.34	106.94 ± 27.51	102.47 ± 24.28	≤0.001	0.634
PLT (10^9^/L)	184.89 ± 44.65	56.28 ± 31.49	85.09 ± 75.73	≤0.001	0.280
AFP (ng/mL)	3.15 ± 1.14	4.32 ± 5.48	7.33 ± 17.28	≤0.001	0.744
Cr (*μ*mol/L)	65.99 ± 12.83	69.31 ± 18.30	90.97 ± 89.62	≤0.001	0.600
FBG (mmol/L)	5.38 ± 0.62	5.28 ± 0.74	8.95 ± 3.85	≤0.001	≤0.001
HBV DNA (IU/mL)					
≤ 10^2^		22 (73.3%)	14 (70%)	—	
10^2^-10^5^	—	4 (13.3%)	3 (15%)		0.968
≥ 10^5^		4 (13.3%)	3 (15%)		
HBeAg					
Positive		8 (26.7%)	8 (40%)		0.322
Negative		22 (73.3%)	12 (60%)		
Antiviral therapy					
Over 1 year	—	22 (73.3%)	14 (70%)	—	0.797
Less than 1 year		8 (26.7%)	6 (30%)		
Child–Pugh					
A	—	8 (26.7%)	6 (30%)	—	
B		19 (63.3%)	12 (60%)		0.966
C		3 (10%)	2 (10%)		
Antidiabetic therapy	—	—	Insulin (*n* = 5)	**—**	**—**
			Acarbose (*n* = 4)		
			Insulin+acarbose (*n* = 1)		
			Metformin (*n* = 1)		
Dietary habit	Mix	Mix	Mix	**—**	**—**

Continuous variables were expressed as means ± standard deviation. BMI: body mass index; WBC: white blood cell; RBC: red blood cell; HGB: hemoglobin; PLT: blood platelet; ALB: albumin; TBil: total bilirubin; ALT: alanine aminotransferase; AST: aspartate aminotransferase; AFP: alpha fetoprotein; Cr: creatinine; FBG: fasting blood glucose; HC: healthy control; LC: HBV-related liver cirrhosis; LCDM: hepatitis B liver cirrhosis and diabetes mellitus.

## Data Availability

The data used to support the findings of this study are available from the corresponding author upon request.

## Abbreviations

ALB: Albumin
ALT: Alanine aminotransferase
AST: Aspartate aminotransferase
AFP: Alpha fetoprotein
BMI: Body mass index
Cr: Creatinine
CTP: Child–Turcotte–Pugh
DM: Diabetes mellitus
EG: Elevated blood glucose
EH: Elevated glycosylated hemoglobin
FBG: Fasting blood glucose
GCP: Good Clinical Practice
HBV: Hepatitis B virus
HC: Healthy controls
HCC: Hepatocellular carcinoma
HGB: Hemoglobin
HBCDI: Hepatitis B cirrhosis dysbiosis indicator
LC: HBV-related liver cirrhosis
LCDM: Hepatitis B liver cirrhosis and diabetes mellitus
LDA: Linear discriminant analysis
LEfSe: Linear discriminant analysis effect size
NH: Normal glycosylated hemoglobin
NG: Normal blood glucose
PLT: Blood platelet
RBC: Red blood cell
TBil: Total bilirubin
WBC: White blood cell

## Supplementary Materials

Supplementary Materials The supplementary file contains Supplementary Figures 1-4.

## References

[1] Lavanchy D. (2004). Hepatitis B virus epidemiology, disease burden, treatment, and current and emerging prevention and control measures. *Journal of Viral Hepatitis*.

[2] Lee W. G., Wells C. I., McCall J. L., Murphy R., Plank L. D. (2019). Prevalence of diabetes in liver cirrhosis: a systematic review and meta-analysis. *Diabetes/Metabolism Research and Reviews*.

[3] Zhang X., Harmsen W. S., Mettler T. A. (2014). Continuation of metformin use after a diagnosis of cirrhosis significantly improves survival of patients with diabetes. *Hepatology: official journal of the American Association for the Study of Liver Diseases*.

[4] Zhao Y., Xing H., Wang X. (2019). Management of diabetes mellitus in patients with chronic liver diseases. *Journal of Diabetes Research*.

[5] Elkrief L., Rautou P. E., Sarin S., Valla D., Paradis V., Moreau R. (2016). Diabetes mellitus in patients with cirrhosis: clinical implications and management. *Liver international: official journal of the International Association for the Study of the Liver*.

[6] Huang Y. W., Wang T. C., Lin S. C. (2013). Increased risk of cirrhosis and its decompensation in chronic hepatitis B patients with newly diagnosed diabetes: a nationwide cohort study. *Clinical Infectious Diseases : An Official Publication of the Infectious Diseases Society of America*.

[7] Bertuccio P., Turati F., Carioli G. (2017). Global trends and predictions in hepatocellular carcinoma mortality. *Journal of Hepatology*.

[8] Tan Y., Wei S., Zhang W., Yang J., Yang J., Yan L. (2019). Type 2 diabetes mellitus increases the risk of hepatocellular carcinoma in subjects with chronic hepatitis B virus infection: a meta-analysis and systematic review. *Cancer Management and Research*.

[9] Blandino G., Inturri R., Lazzara F., Di Rosa M., Malaguarnera L. (2016). Impact of gut microbiota on diabetes mellitus. *Diabetes & Metabolism*.

[10] Lu H., Wu Z., Xu W., Yang J., Chen Y., Li L. (2011). Intestinal microbiota was assessed in cirrhotic patients with hepatitis B virus infection. Intestinal microbiota of HBV cirrhotic patients. *Microbial Ecology*.

[11] Sun X., Pan C. Q., Xing H. (2021). Effect of microbiota metabolites on the progression of chronic hepatitis B virus infection. *Hepatology International*.

[12] Wei X., Yan X., Zou D. (2013). Abnormal fecal microbiota community and functions in patients with hepatitis B liver cirrhosis as revealed by a metagenomic approach. *BMC Gastroenterology*.

[13] Chen Z., Radjabzadeh D., Chen L. (2021). Association of insulin resistance and type 2 diabetes with gut microbial diversity: a microbiome-wide analysis from population studies. *JAMA Network Open*.

[14] Yixuan W., Shan Z., Danying C., Shunai L., Jing S., Huichun X. (2019). Correlation between hepatitis B cirrhosis dysbiosis indicator and severity of hepatitis B cirrhosis. *Chinese Journal of Liver Diseases (Electronic Version)*.

[15] Qin J., Li R., Raes J. (2010). A human gut microbial gene catalogue established by metagenomic sequencing. *Nature*.

[16] Cani P. D., Van Hul M., Lefort C., Depommier C., Rastelli M., Everard A. (2019). Microbial regulation of organismal energy homeostasis. *Nature Metabolism*.

[17] Levy M., Blacher E., Elinav E. (2017). Microbiome, metabolites and host immunity. *Current Opinion in Microbiology*.

[18] Gurung M., Li Z., You H. (2020). Role of gut microbiota in type 2 diabetes pathophysiology. *eBioMedicine*.

[19] Arab J. P., Martin-Mateos R. M., Shah V. H. (2018). Gut-liver axis, cirrhosis and portal hypertension: the chicken and the egg. *Hepatology International*.

[20] Zhang J., Guo Z., Xue Z. (2015). A phylo-functional core of gut microbiota in healthy young Chinese cohorts across lifestyles, geography and ethnicities. *The ISME Journal*.

[21] Vital M., Howe A. C., Tiedje J. M. (2014). Revealing the bacterial butyrate synthesis pathways by analyzing (meta) genomic data. *MBio*.

[22] Wu H., Tremaroli V., Schmidt C. (2020). The gut microbiota in prediabetes and diabetes: a population-based cross-sectional study. *Cell Metabolism*.

[23] Candela M., Biagi E., Soverini M. (2016). Modulation of gut microbiota dysbioses in type 2 diabetic patients by macrobiotic Ma-Pi 2 diet. *The British Journal of Nutrition*.

[24] Zhong H., Ren H., Lu Y. (2019). Distinct gut metagenomics and metaproteomics signatures in prediabetics and treatment-naive type 2 diabetics. *eBioMedicine*.

[25] Pinna N. K., Anjana R. M., Saxena S. (2021). Trans-ethnic gut microbial signatures of prediabetic subjects from India and Denmark. *Genome Medicine*.

[26] Lê K. A., Li Y., Xu X. (2013). Alterations in fecal Lactobacillus and Bifidobacterium species in type 2 diabetic patients in Southern China population. *Frontiers in Physiology*.

[27] Anhê F. F., Barra N. G., Cavallari J. F., Henriksbo B. D., Schertzer J. D. (2021). Metabolic endotoxemia is dictated by the type of lipopolysaccharide. *Cell Reports*.

[28] de la Cuesta-Zuluaga J., Mueller N. T., Corrales-Agudelo V. (2017). Metformin is associated with higher relative abundance of mucin-degrading Akkermansia muciniphila and several short-chain fatty acid-producing microbiota in the gut. *Diabetes Care*.

[29] Long B. W. L. K. J., Lu R. F. M., Han T. (2018). Structure of intestinal microflora in hepatitis B cirrhosis patients and hepatitis B cirrhosis patients with diabetes mellitus. *Shijie Huaren*.

[30] Loomba R., Ling L., Dinh D. M. (2021). The commensal microbe Veillonella as a marker for response to an FGF19 analog in NASH. *Hepatology: official journal of the American Association for the Study of Liver Diseases*.

[31] Poppleton D. I., Duchateau M., Hourdel V. (2017). Outer membrane proteome of Veillonella parvula: a diderm firmicute of the human microbiome. *Frontiers in Microbiology*.

[32] Tamanai-Shacoori Z., Smida I., Bousarghin L. (2017). Roseburia spp.: a marker of health?. *Future Microbiology*.

[33] Belcheva A., Irrazabal T., Robertson S. J. (2014). Gut microbial metabolism drives transformation of MSH2-deficient colon epithelial cells. *Cell*.

[34] Zhang C., Wang C., Li S. (2022). Meta-analysis of randomized controlled trials of the effects of probiotics on type 2 diabetes in adults. *Clinical nutrition: official journal of the European Society of Parenteral and Enteral Nutrition*.

[35] Bajaj J. S., Heuman D. M., Hylemon P. B. (2014). Randomised clinical trial: Lactobacillus GG modulates gut microbiome, metabolome and endotoxemia in patients with cirrhosis. *Alimentary Pharmacology & Therapeutics*.

