# Supplementary material for: Characteristics and Clinical Significance of Intestinal Microbiota in Patients with Chronic Hepatitis B Cirrhosis and Type 2 Diabetes Mellitus
Source: J Diabetes Res. 2022 May 13;2022:1826181. doi: 10.1155/2022/1826181 (PMC9122699; doi:10.1155/2022/1826181)

**Characteristics and clinical significance of intestinal** **microbiota in patients with** **chronic hepatitis B cirrhosis and type 2 diabetes** **mellitus**

Xiu Sun^1^, Xin Chi^1^,Yingying Zhao^1,3^, Shunai Liu^2,3^, Huichun Xing^1,3,4^

1. Center of Liver Diseases Division 3, Beijing Ditan Hospital, Capital Medical University, Beijing, China

2. Beijing Key Laboratory of Emerging Infectious Diseases, Institute of Infectious Disease, Beijing Ditan Hospital, Capital Medical University, Beijing, China

3. National Center For Infectious Diseases，Beijing, China

4. Peking University Ditan Teaching Hospital, Beijing, China

Supplementary Figure 1：The relative abundance of intestinal microbiota in HC, NG and EG at the phylum level


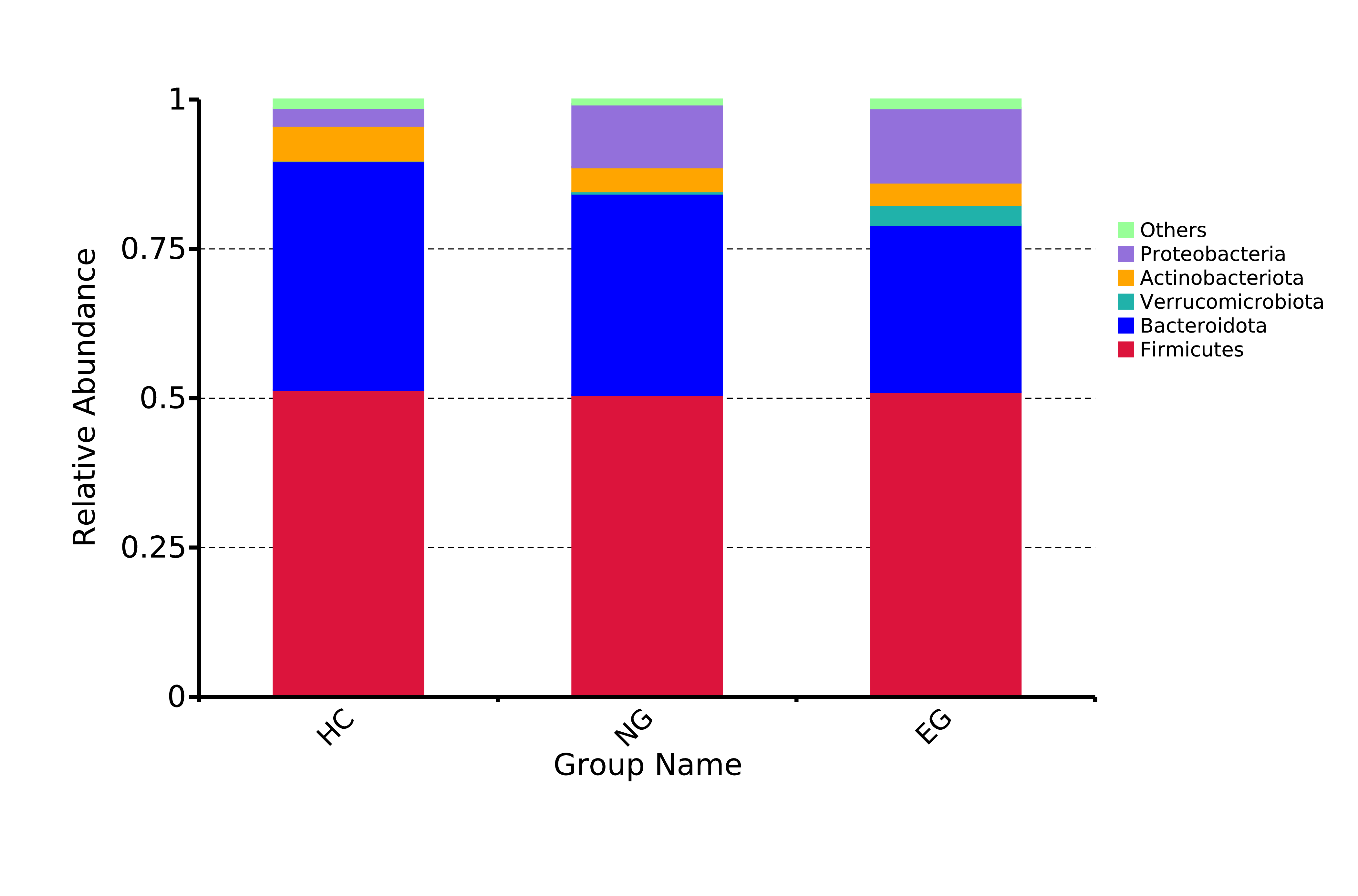


Supplementary Figure 2：The relative abundance of intestinal microbiota in HC, NG and EG at the genus level


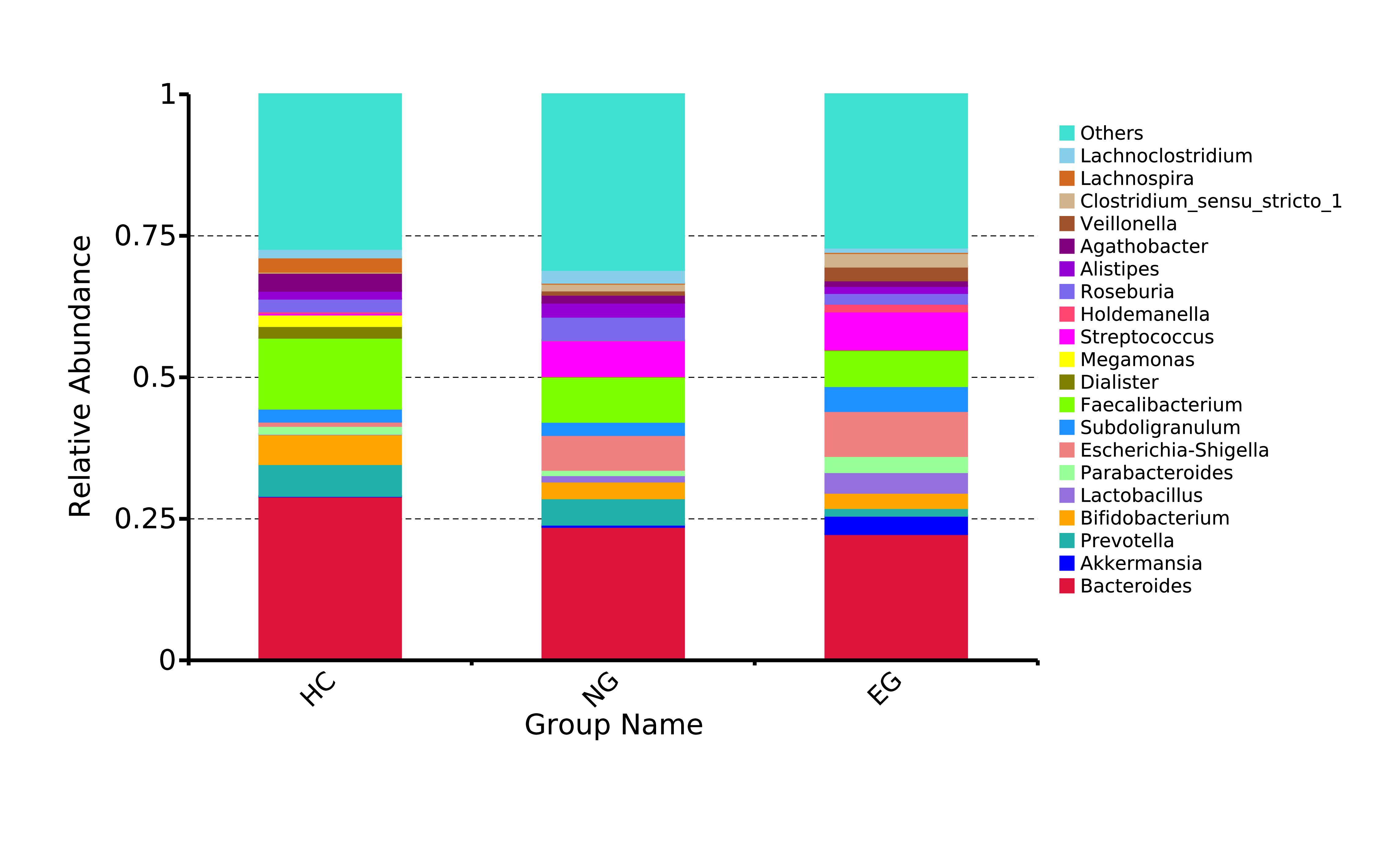


Supplementary Figure 3：The relative abundance of intestinal microbiota in HC, NH and EH at the phylum level


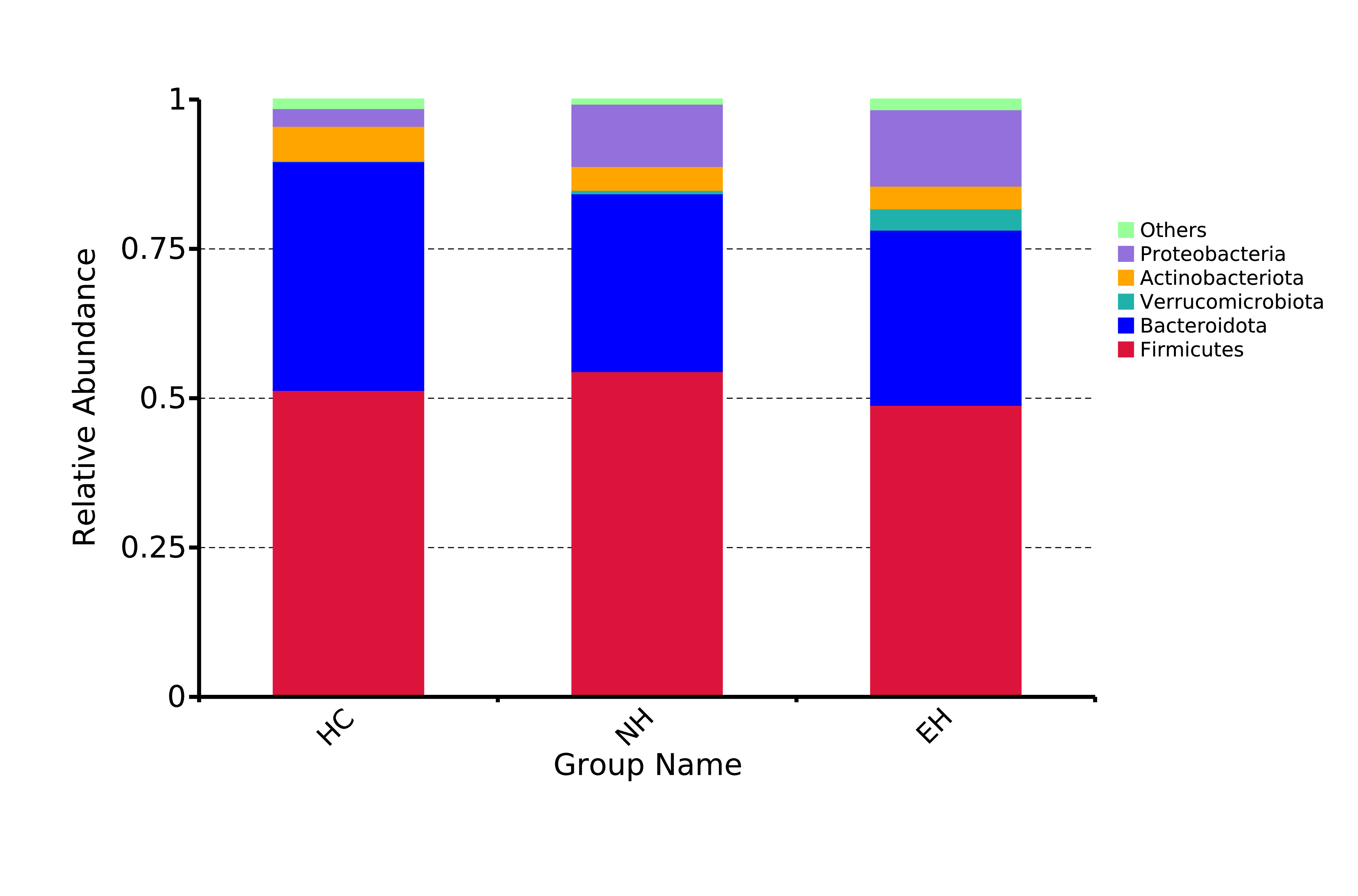


Supplementary Figure 4：The relative abundance of intestinal microbiota in HC, NH and EH at the genus level


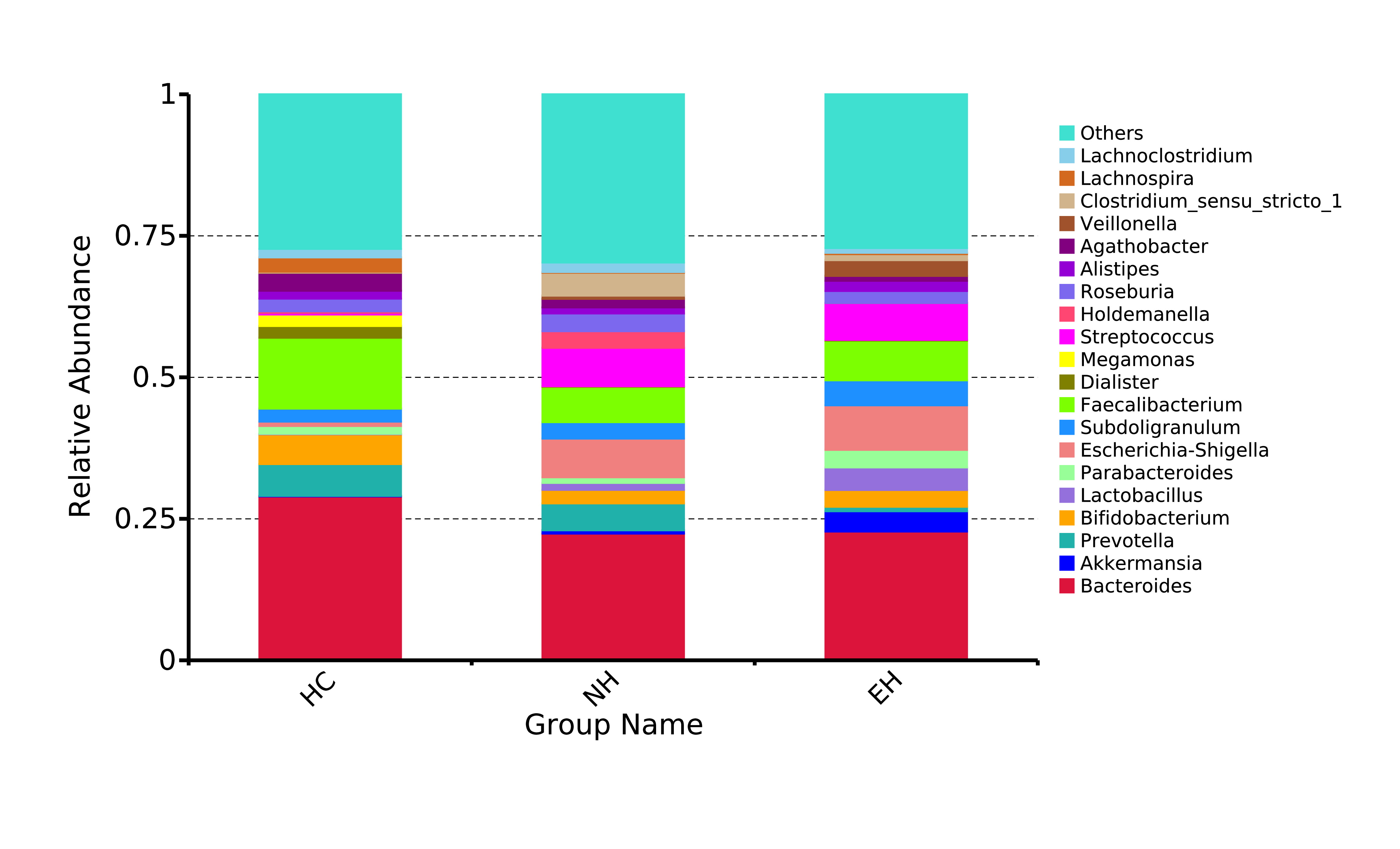

Supplement: Supplementary Materials — The supplementary file contains Supplementary Figures 1-4. [file 1826181.f1.doc]
